# Psychotic denial of pregnancy: case report and narrative literature review.

**DOI:** 10.1192/j.eurpsy.2023.2401

**Published:** 2023-07-19

**Authors:** M. Martín Velasco, I. Romero Gerechter, C. Díaz Mayoral, E. Arroyo Sánchez, A. Sanz Giancola, P. Setién Preciados

**Affiliations:** 1 Psychiatry; 2 Hospital Universitario Príncipe de Asturias, Alcalá de Henares, Spain

## Abstract

**Introduction:**

Denial of pregnancy is the phenomenon where a woman fails to recognize or accept her pregnancy at >20 weeks gestational age. It associates with increased morbidity and mortality of mother and child, and can be classified as non-psychotic or psychotic. There is fewer medical literature regarding the latter, making it difficult to recognize, let alone to treat, since we do not have robust data regarding incidence nor approved interventions or treatment.

**Objectives:**

To describe this unfamiliar entity in order to be able to perform a proper diagnosis and thus prevent possible negative outcomes.

**Methods:**

We present a case report alongside a narrative literature review on the topic.

**Results:**

We report the case of a 39-year-old caucasian woman, foreign, undomiciled, with an advanced pregnancy, who was admitted to a Psychiatry in-patient unit due to psychotic symptoms such as mistrust and delusions. She showed scarce collaboration during assessment and did not give any plausible information about her identity. Her birthplace and prior medical records were therefore unknown. Apparently, she had no family nor social support network. Despite the obvious signs, she did not recognize being pregnant and showed great irritability when asked; her responses ranged from claiming she was suffering from a gastric tumor and making delusional attributions of symptoms clearly related to the pregnancy to partially acknowledging her state but refusing to answer any questions on the matter. Blood work showed no significant abnormalities and obstetric ultrasound revealed a low risk 35 weeks pregnancy.

With an estimated prevalence of 1:475 in general population, denial of pregnancy is not as rare as it may seem. The psychotic variant, however, is rather uncommon. Typically, women with psychotic pregnancy denial have prior history of major mental illness, most frequently schizophrenia, and suffer from extreme psychosocial vulnerability. They usually present previous or anticipated child custody loss, which hampers the process of developing antenatal attachment behaviours. Psychotic denial does not associate with concealing, since these women are mentally detached from the gestation and tend to create delusional explanations to their pregnancy symptoms. Not all of them show complete denial, some being able to acknowledge it, though mostly in an inconsistent way. These patients often fail to seek prenatal care or are noncompliant, they are at greater risk of drug exposure, and some are unable to recognize symptoms of labour, all of which increases the rate of negative outcomes for mother and baby, including neonaticide.

**Conclusions:**

Psychotic denial is a rare diagnosis which should be properly assessed due to its clinical implications and the need to prevent potential negative outcomes for mother and baby.

**Disclosure of Interest:**

None Declared

